# ﻿Two new species of the mealybug genus *Paraputo* Laing (Hemiptera, Pseudococcidae) from Borneo Island, Indonesia

**DOI:** 10.3897/zookeys.1249.159569

**Published:** 2025-08-13

**Authors:** Agustin Zarkani, Habib Al Ayubi Reonaldi, Ariffatchur Fauzi, Dwinardi Apriyanto, Yahumri, Rosfiansyah, Mehmet Bora Kaydan

**Affiliations:** 1 Department of Plant Protection, Faculty of Agriculture, University of Bengkulu, 383711, Bengkulu, Sumatra, Indonesia University of Bengkulu Bengkulu Indonesia; 2 Research Organization for Agriculture and Food, The National Research and Innovation Agency of Indonesia, Cibinong, 16911, Jawa Barat, Java, Indonesia Research Organization for Agriculture and Food, The National Research and Innovation Agency of Indonesia Cibinong Indonesia; 3 Department of Agroecotechnology, Faculty of Agriculture, Mulawarman University, Samarinda, 75123, East Kalimantan, Indonesia Mulawarman University Samarinda Indonesia; 4 Biotechnology Development and Research Centre, Çukurova University, 01250, Adana, Turkiye Çukurova University Adana Turkiye

**Keywords:** Biodiversity, Coccomorpha, identification key, insect pests, morphology, Sternorrhyncha, taxonomy

## Abstract

The mealybug genus *Paraputo* Laing (Hemiptera: Coccomorpha: Pseudococcidae) contains 89 species worldwide, including several important agricultural insect pests. During a 2024 survey of mealybug species on Borneo Island, Indonesia, two undescribed *Paraputo* species were found feeding on forest trees. Descriptions of both new species, based on morphological characters, are accompanied by photomicrographs and taxonomic illustrations. The newly described species, *Paraputoraufi* Zarkani & Kaydan, **sp. nov.** and *Paraputomartonoi* Zarkani & Kaydan, **sp. nov.**, are each compared with other species of *Paraputo* to which they show notable morphological similarities. An updated taxonomic key to species of *Paraputo* found in Southeast and southern Asia is provided.

## ﻿Introduction

Worldwide, mealybugs (Hemiptera: Coccomorpha: Pseudococcidae) are widely known as significant agricultural pests (Daane et al. 2012; Zarkani et al. 2021). They produce a mealy wax that forms a protective layer over their bodies, sometimes also covering large colonies and even extending onto nearby surfaces of the host plant (Cox and Pearce 1983). These sap-sucking insects not only cause direct damage to plants by extracting sap but also have the potential to transmit various plant viruses (Daane et al. 2012; Ahmed et al. 2023). Infested plants may show symptoms such as chlorosis, stunted growth, and the drying of seed pods, branches, and twigs, and in cases of severe infestation, entire plants may decline or die (Daane et al. 2012). In addition, mealybugs produce sugary honeydew waste, which serves as an ideal medium for sooty mold growth. The mold blocks light and air from the plant, interfering with photosynthesis and accelerating plant deterioration. While most mealybug species are polyphagous, often being capable of feeding on over a hundred plant species (Ahmed et al. 2023), some have a more restricted diet, being oligophagous (Francardi and Covassi 1992; Franco et al. 2009) or even monophagous (Ben-Dov 1994).

Around 2065 mealybug species have been described, classified under 264 genera (García-Morales et al. 2016). About 89 species have been documented in the genus *Paraputo* worldwide, with 13 of these species recorded in Indonesia (Watson et al. 2014; Zarkani et al. 2021, 2023a, b, 2024a, b). Nine of them, namely *Paraputoacehicus* Williams, *P.mangiferae* (Betrem), *P.martini* Williams, *P.maschwitzi* Williams, *P.moogi* Williams, *P.neonaucleae* Williams, *P.odontomachi* (Takahashi), *P.palmicola* Williams, *P.pandanicola* Williams and *P.riparius* Williams, are endemic and mono/oligophagous species in Indonesia (Williams 2004; Zarkani et al. 2021). Many of the other species are cosmopolitan, polyphagous and much more widespread.

Adult females of *Paraputo* can be readily distinguished from other mealybug genera by several morphological features: (i) the body is broadly oval to rounded and membranous; (ii) the cerarii are either present in a continuous marginal zone or are distinct, with the number ranging from 11–18 pairs (specimens from southern Asia) or 5–18 pairs (in specimens from elsewhere); and (iii) discoidal pores, if present, are scattered and are either heavily sclerotized with reticulated surfaces or form distinct dorsal submarginal and medial groups (Williams and Granara de Willink 1992; Williams 2004; Danzig and Gavrilov 2010).

Members of *Paraputo* share several morphological features with species of *Formicococcus* Takahashi, such as the broadly oval to rounded body of the adult female, stout legs, prominent ostioles, and cerarii each containing numerous conical setae. Nevertheless, female *Formicococcus* can generally be distinguished from those of *Paraputo* by having an anal lobe bar, a structure absent from *Paraputo* species. Additionally, some *Paraputo* species bear a close resemblance to those in the genus *Dysmicoccus* Ferris, by having sclerotized areas on the ventral surfaces of the anal lobes and the presence of multiple conical setae in the cerarii. However, a key difference between these genera lies in the leg segment proportions; in *Dysmicoccus*, the combined length of the tibia and tarsus typically exceeds that of the trochanter and femur, whereas in many *Paraputo* species, it is distinctly shorter (Williams 2004; Joshi et al. 2024). Williams (2004) distinguished these two genera primarily by the presence or absence of the anal lobe bar. However, Danzig and Gavrilov-Zimin (2015) disagreed, arguing that the presence of the anal lobe bar may vary among individuals; they instead proposed distinguishing the genera based on the number of setae on the anal ring. Here, this work follows Williams (2004), who considered the number of anal ring setae to be subject to intraspecific variation and so lacking generic significance. Our observations show that the anal ring typically has six primary setae, and when additional setae are present, they are generally slender, short and variable in number and position.

In this study, the adult females of two new *Paraputo* species are described based on morphological characters, accompanied by photomicrographs and taxonomic illustrations. An updated taxonomic key to species of *Paraputo* found in Southeast and southern Asia is provided.

## ﻿Material and methods

Adult female mealybugs were collected during multiple sampling events between March and December 2024, from the leaves, trunks, and branches of forest trees on Borneo Island, Indonesia. The sampling locations ranged in elevation from sea level up to 1100 meters. Slide mounts of adult female specimens were prepared with the aid of a LEICA EZ4HD binocular dissection microscope following the method described by Kosztarab and Kozár (1988), except that specimens were alternately heated at 50 °C and 80 °C, each for 30 minutes, for a total duration of up to 2 hours or more as needed.

Species identification was carried out by examining diagnostic morphological features of the adult female mealybugs using a phase-contrast compound microscope (LEICA DM2700). Identifications were made using the keys provided in Williams and Watson (1988), Williams and Granara de Willink (1992), and Williams (2004). The morphological criteria applied were based on the standards set by Williams (2004) and Zarkani et al. (2023b). The measurements are given first for the holotype, followed by the full range for all specimens in parentheses. Body length and width were measured in millimeters (mm), representing the longest longitudinal and widest transverse dimensions, respectively. All other anatomical measurements were recorded in micrometers (μm). Total antennal length was calculated as the sum of all the segments, while total leg length comprised the combined lengths of the trochanter + femur, tibia + tarsus, coxa and claw. In the accompanying taxonomic illustrations, dorsal structures are depicted on the left side and ventral structures on the right. Type specimens of the new species described have been deposited in the Mealybugs Museum, Department of Plant Protection, Faculty of Agriculture, University of Bengkulu, Bengkulu, Sumatra, Indonesia (**MMUB**), and at Museum Zoologicum Bogoriense, Bogor, West Java, Indonesia (**MZB**). In the listing of holotype data under material examined, the line breaks on the slide labels are indicated using “/”.

## ﻿Results and discussion

### ﻿Genus Paraputo

Taxon classificationAnimaliaHemipteraPseudococcidae

Laing

F4F525B0-C7DA-59BE-B72B-AEABB0471088

#### Type species.

*Lachnodiopsisszemaoensis* Borchsenius, by original designation.

#### Generic diagnosis

**(adapted from Williams (2004).** Body of adult female broadly oval to rounded, membranous. Antennae each with 6 to 8 antennomeres. Legs well developed, generally robust, femur often about twice as wide as tibia. Combined length of tibia and tarsus usually shorter than that of trochanter and femur. Translucent pores commonly present on hind coxae, sometimes also on hind femur and tibia, and occasionally also on coxae of middle legs. Claws stout and lacking a denticle. Labium typically elongate, usually longer than clypeolabral shield. Anal ring generally located on dorsum at a distance equal to or greater than its length from apex of abdomen and bearing six or more setae. Circulus well developed or absent. Cerarii numbering 11 to 18 pairs; those on anal lobes and posterior abdominal segments each usually containing multiple conical setae, sometimes accompanied by normal setose setae that are often of similar basal width to conical setae, along with clusters of trilocular pores. In rare cases, cerarii each reduced to only two conical setae; often with intermediate cerarii or conical setae present also, thus forming a continuous row of conical setae around dorsal margin, frequently accompanied by a continuous band of densely packed trilocular pores; conical setae rarely present on ventral margin. Ostioles prominent, situated away from body margins, each wide, with inner lip edges sclerotized; each lip often with abundant trilocular pores, rarely with few, and bearing few to numerous setae. Eyes present. Spiracles large and conspicuous.

***Dorsum*** with setae usually numerous, either minute and stiff or longer and flagellate, often with longer setae flanking anal ring. Trilocular pores abundant on both surfaces. Multilocular disc pores and oral collar tubular ducts absent.

***Venter*** usually with flagellate setae, sometimes resembling those on dorsum. Cisanal and obanal setae usually apparent, sometimes each long and stout and displaced onto dorsum posterior to anal ring. Oral collar tubular ducts present, usually of only 1 size, either 1.5–2.0 times wider than a trilocular pore (referred to as large type), or as wide as, or narrower than a trilocular pore; but sometimes 2 or 3 different sizes present. Tubular ducts sometimes present across medial area and in marginal groups on abdomen, sometimes also present between antennal bases; present or absent between anal lobes. Anal lobes each either membranous or with various degrees of sclerotization, at times sclerotization occupying most of lobe, but never with a distinct anal lobe bar; occasionally ventral margins of some anterior abdominal segments also sclerotized (Williams and Granara de Willink 1992; Williams 2004; Danzig and Gavrilov 2010).

### ﻿ Paraputo martonoi

Taxon classificationAnimaliaHemipteraPseudococcidae

Zarkani & Kaydan sp. nov.

FCD7B2A8-1669-511A-A889-9B7C3D6969E0

https://zoobank.org/3CEFE0BF-0AB2-407E-9105-28FD04721305

Figs 1 , 2 , 3

#### Material examined.

All deposited at MMUB. ***Holotype*.** Adult female, left label: AZ3100 / 15.xi.2024 / Indonesia, East Kalimantan Province, Sepaku / Rubiaceae / 0°55'34.7"S / 116°45'16.0"E / 30 m a.s.l; right label: *Paraputomartonoi* Zarkani & Kaydan, 1♀ / coll. A. Zarkani / det. M.B. Kaydan. In addition to the holotype specimen (ringed with red ink on the coverslip), the slide mount also contains 2 specimens of *P.martonoi* (MMUB). ***Paratypes*.** Indonesia: same data as for holotype; • 3 ♀♀ on one slide, each slide with 2 specimens (AZ3101) at MZB.

#### Description of adult female.

**Figure 1.**
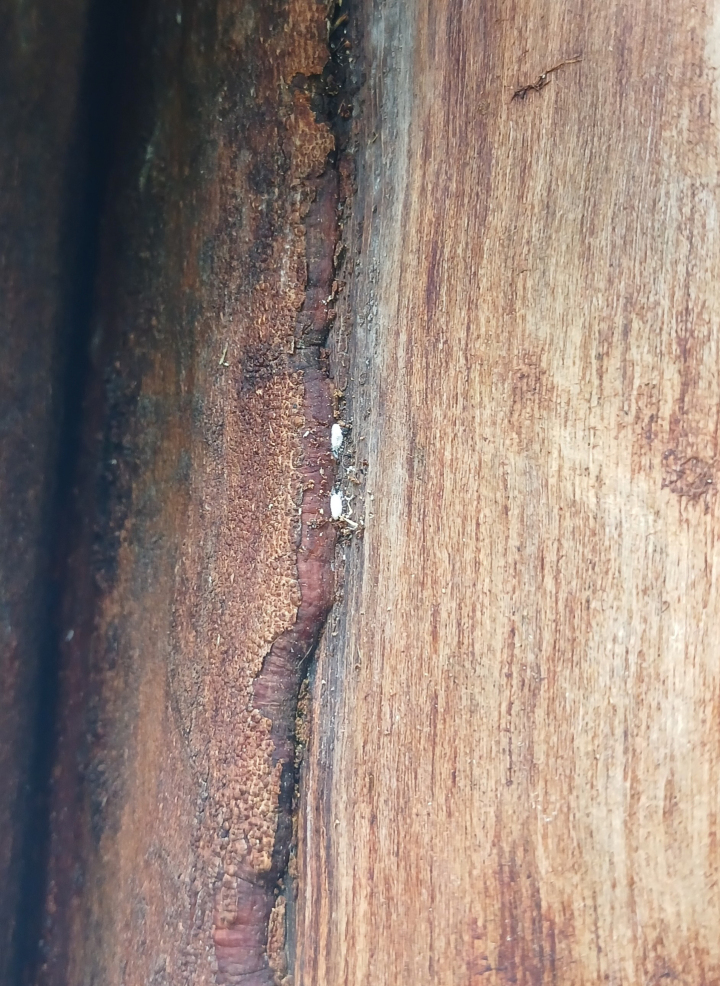
Unidentified host plant (Rubiaceae) of *Paraputomartonoi* Zarkani & Kaydan, sp. nov.; the mealybugs live within ants’ nests inside the branches.

***Appearance in life*.** Adult females produce a powdery white wax covering the dorsal surface of the body. Living on woody parts of its host plant (Fig. 1), commonly attended by ants of the genus *Dolichoderus* Lund (Formicidae) (not visible in Fig. 1).

**Figure 2.**
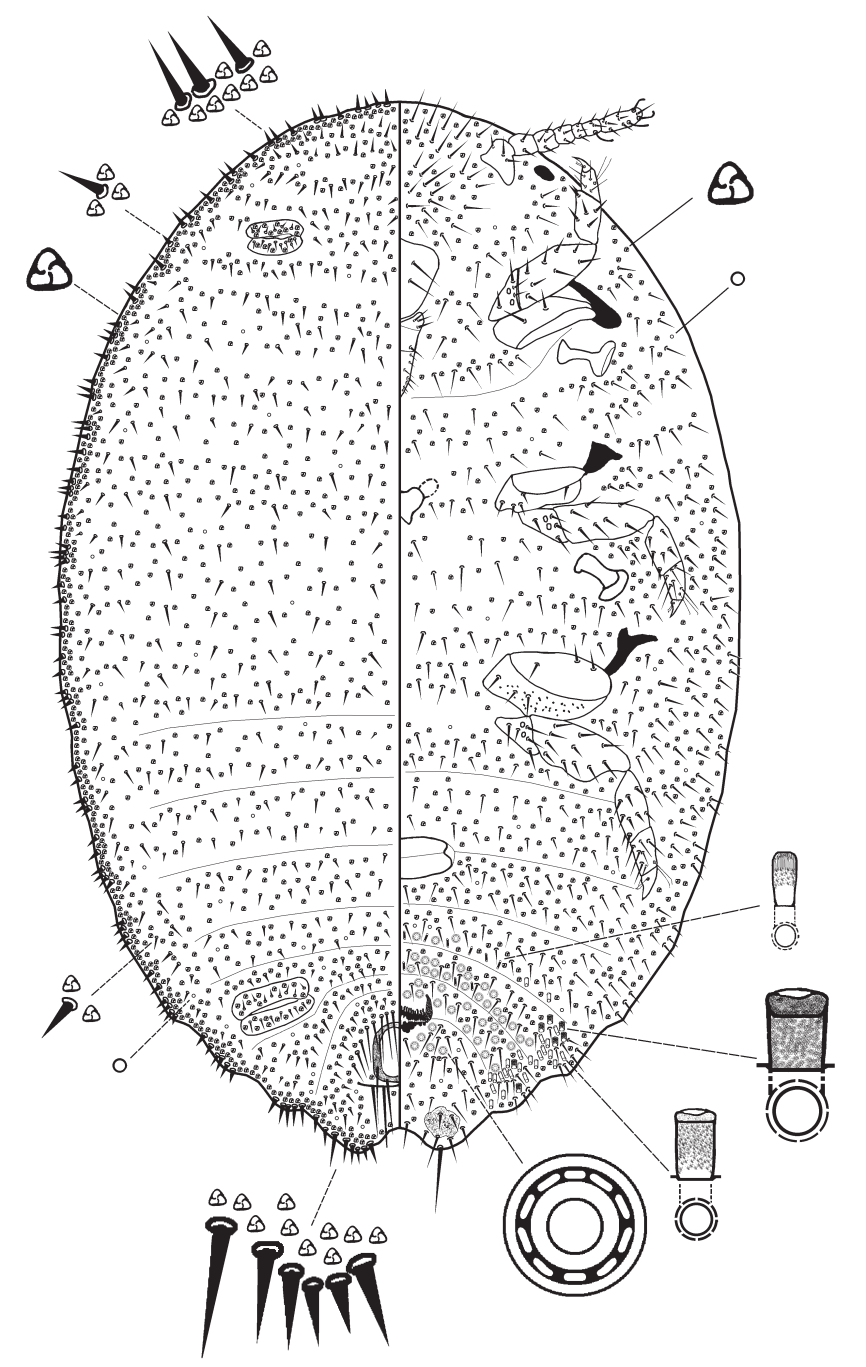
Adult female of *Paraputomartonoi* Zarkani & Kaydan, sp. nov., holotype.

**Figure 3.**
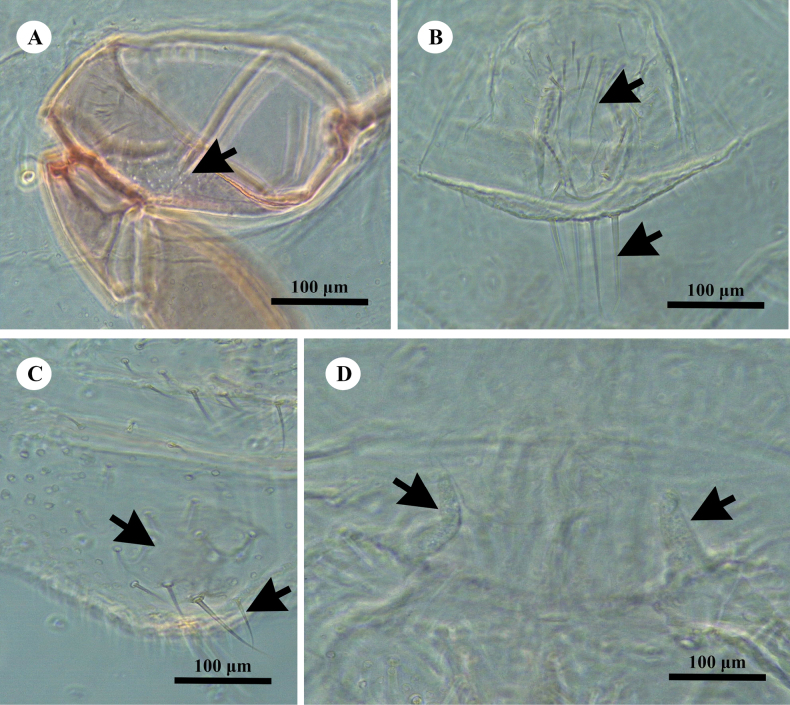
*Paraputomartonoi* Zarkani & Kaydan, sp. nov., adult female holotype. A. Stout legs; B. Translucent pores (few) present on hind coxa only; C. Dorsal setae slender and stiff, each about 20–25 µm long; D. Ventral sclerotized patches present on margins of abdominal segments VI–VIII; E. Multilocular disc pores present by posterior medial edge of abdominal segment VII only.

***Slide-mounted adult female*** (values of holotype followed by range of 3 paratypes in parentheses) (Figs 2, 3). Body broadly oval, sometimes almost rotund, membranous, largest specimens 2.13 (2.13–2.25) mm long and 1.62 (1.62–1.73) mm wide. Anal lobes weakly developed, each ventral surface bearing a stout apical seta 80 (80–88) μm long arising from a large circular-to-oval sclerotized area, about 57–79 μm wide, on ventral margin of abdominal segment VIII; small sclerotized areas present also on ventral margins of segments VII and VI. Antennae each 340 (340–360) μm in total length, with 7 antennomeres; antennal setae mostly short. Individual antennal antennomere lengths (in μm) are as follows: antennomere I–VI, each 260 (260–270); and VII (apical antennomere), 80 (80–90) long and 40 (40–45) wide. Apical antennomere with 4 fleshy setae, each 30–38 μm long, and an apical seta 36 (36–90) μm long. Clypeolabral shield 258 (258–263) μm long and 245 (245–250) μm wide. Labium unusually long and pointed, longer than clypeolabral shield, 3 segmented, 280 (280–300) μm long, with a basal segment 150 (150–155) μm wide. Anterior spiracles each 110 (110–113) μm long and 58 (58–60) μm wide across the atrium, while the posterior spiracles are 110 (110–113) μm long and 65 (50–65) μm wide across the atrium. Legs well developed (Fig. 3A); hind leg segments measuring (in μm): coxa, 213 (213–215); trochanter+femur, 378 (378–400); tibia+tarsus, 238 (238–253); and claw, 55 (55–70), without a denticle. Ratio of lengths of hind tibia+tarsus to trochanter+femur 0.69 (0.69–0.7): 1; ratio of lengths of the tibia to tarsus 1.5 (1.2–1.5): 1; and ratio of length of trochanter+femur to the greatest width of femur 3.1 (3.1–3.2): 1. Hind legs with only about 10–20 translucent pores, by anterior margin of coxa only (Fig. 3B). Tarsal digitules setose, each 48–50 μm long, claw digitules also setose, each about 28–30 μm long. Anterior and posterior ostioles well developed, each containing a total of 62 (60–70) trilocular pores across both lips and 14 (14–25) setae. Circulus situated between abdominal segments III and IV, 140 (140–150) μm wide and divided by an intersegmental line. Anal ring about 108 (100–108) μm wide, situated on dorsum at about 1.0–1.5 times its length from apex of abdomen; with 2 rows of cells and bearing 6 setae, each 78–83 μm long. Cerarii numbering not distinct, with many intermediate conical setae, merged to form a marginal band of trilocular pores and enlarged conical setae. Anal lobe cerarii each normally containing 6 or 7 conical setae of different sizes, largest about 25 μm long and 7.5 μm wide at base, and a small compact group of trilocular pores. Anterior cerarii similar to anal lobe cerarii but with largest conical setae slenderer, and each cerarius often subdivided into 2 or 3 smaller cerarii: additional conical setae also present, so that cerarii appear to form a continuous band around margin.

***Dorsum*** with slender, stiff setae, each about 20–25 µm long, present around margins and across abdominal segments (Fig. 3C). Setae flanking anal ring longer than other dorsal setae, longest 78–88 µm long but mostly each 45–50 µm long, each seta shorter than or same length as an anal ring seta (each ring seta about 75–88 µm long). Apparent cisanal and obanal setae stout, each 100–113 µm long, displaced onto dorsum, situated immediately posterior to anal ring. Multilocular disc pores absent. Trilocular pores abundant, evenly distributed. Discoidal pores fairly numerous, each only slightly smaller than a trilocular pore.

***Venter*** with normal flagellate setae present, mostly slender and each longer than a dorsal seta, each 33–58 µm long. Ventral sclerotized patches present on anal lobes and on margins of abdominal segments VI and VII (Fig. 3D). Multilocular disc pores, each 7.5–8.8 μm in diameter, few, numbering only 6–10 by posterior medial edge of abdominal segment VII only (Fig. 3E). Trilocular pores numerous but less abundant than on dorsum. Discoidal pores same size as on dorsum, scattered. Vulva becoming heavily sclerotized at maturity, 100–113 μm long and 88–100 μm wide. Trilocular pores same as those on dorsum, each 2.5–3.8 μm wide, scattered throughout. Oral collar tubular ducts of 2 sizes: larger ducts each 10–13 μm long and 3.8 μm wide, few, with 2 or 4 situated marginally on abdominal segments VII and VIII; smaller ducts each 7–8 μm long and 2.5 μm wide, intermingled with a few large ducts, forming a submarginal-to-marginal cluster of 10–14 ducts on each marginal area between segments VII and VIII, a few also present in medial area of abdominal segment VI.

#### Etymology.

The species is named after Prof. Dr. Edhi Martono, an Indonesian entomologist who has dedicated his work to Integrated Pest Management (IPM) and the toxicology of insecticides.

#### Host plants.

Unidentified forest tree (Rubiaceae) (Fig. 1).

#### Distribution.

Indonesia (Kalimantan, East Kalimantan Province, Sepaku).

#### Comments.

*Paraputomartonoi* is most similar to *P.carnosae* (Takahashi) in having: (i) large oral collar tubular ducts on abdomen only, each over 1.5 times as wide as a trilocular pore; (ii) a large circulus divided by an intersegmental line; (iii) setae on each side of anal ring, of similar length or a bit shorter than an anal ring seta, and ventral submarginal setae on abdominal segments V–VIII shorter than an anal ring seta; and (iv) ventral sclerotized patches present on anal lobes, also on ventral margins of abdominal segment VII, and sometimes VI. However, *P.martonoi* can be readily distinguished from *P.carnosae* by having (character state for *P.carnosae* given in parentheses): (i) dorsal surface with slender, stiff setae, each about 20–25 µm long (with minute setae, stiff and pointed, mostly each about 7.5 µm long); (ii) legs stout (slender); (iii) translucent pores present on hind coxa only (translucent pores present on hind coxa and tibia); (iv) oral collar tubular ducts present on abdominal segments VII and VIII (present on segments V–VIII); and (v) multilocular disc pores present by posterior medial edge of abdominal segment VII only (present by posterior medial edges of segments VI–IX).

*Paraputomartonoi* also resembles *P.latebrae* Williams in possessing large oral collar tubular ducts present between anal lobes. However, *P.martonoi* can be readily distinguished by having (character states for *P.latebrae* given in parenthesis): (i) large oral collar tubular ducts absent on head (present on head, usually between antennal bases); (ii) ventral sclerotized patches present on margins of abdominal segments VI and VII (present only on margins of abdominal segment VII); (iii) cisanal setae slender, much shorter than anal ring setae, each 20–30 µm long (cisanal setae stout, similar to anal ring setae and about same length, each 70–75 µm long); and (iv) multilocular disc pores present on medial edge of abdominal segment VII only (present across medial areas of abdominal segments VI and VII and sometimes represented on segment V and vulva).

### ﻿ Paraputo raufi

Taxon classificationAnimaliaHemipteraPseudococcidae

Zarkani & Kaydan sp. nov.

AC2F415A-D19F-5996-9EC9-2D79F71F03DE

https://zoobank.org/1494A21D-8A34-4A76-BF41-F6B454310B31

Figs 4 , 5 , 6

#### Material examined.

***Holotype*.** Adult female, left label: AZ3102 / 15.xi.2024 / Indonesia, East Kalimantan Province, Sepaku / Meliaceae / 0°58'05.4"S / 116°42'55.3"E / 30 m a.s.l; right label: *Paraputoraufi* Zarkani & Kaydan, 1♀ / coll. A. Zarkani / det. M.B. Kaydan. In addition to the holotype specimen (ringed with red ink on the coverslip), the slide mount also contains 2 specimens of *P.raufi* (MMUB). ***Paratypes*.** Indonesia: same data as for holotype; • 3 ♀♀ on one slide, each slide with 2 specimens (AZ3103) at MZB.

#### Description of adult female.

**Figure 4.**
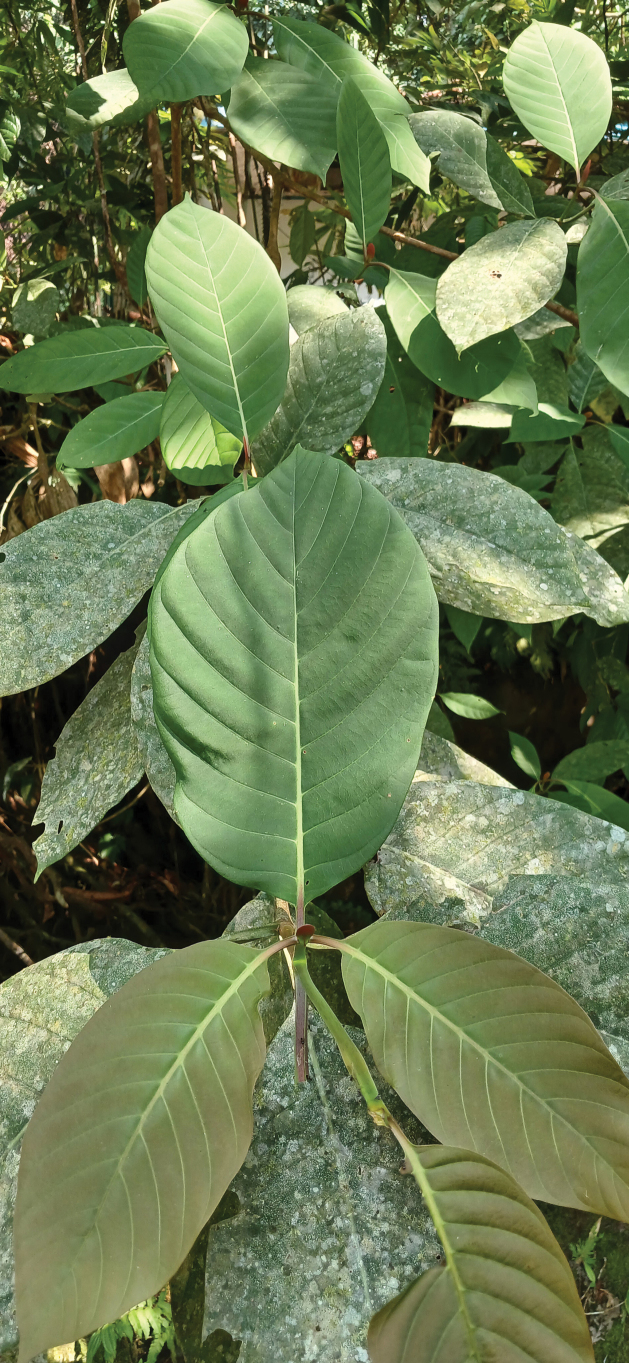
Aggregation of *Paraputoraufi* Zarkani & Kaydan, sp. nov., on the damaged trunk of an unidentified forest tree (Meliaceae).

***Appearance in life*** (Fig. 4). The adult female secretes powdery white wax covering over the dorsal surface of the body. Living on woody parts of the host plant, commonly attended by ants of the genus *Dolichoderus* Lund (Formicidae).

**Figure 5.**
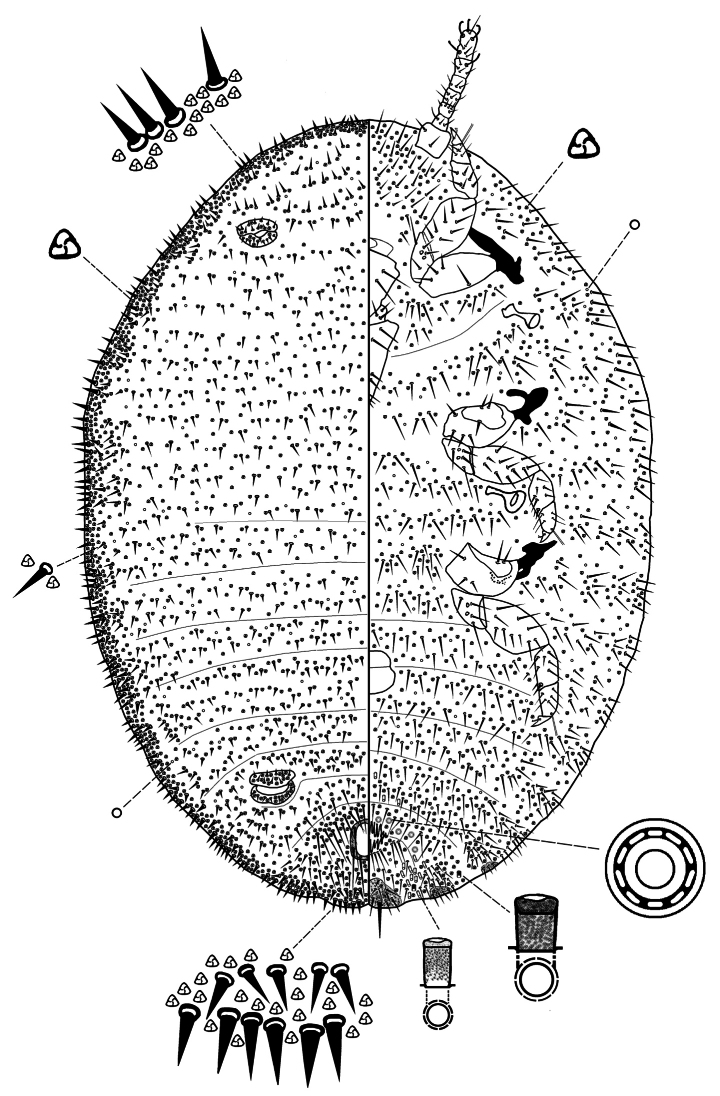
Adult female of *Paraputoraufi* Zarkani & Kaydan, sp. nov., holotype.

**Figure 6.**
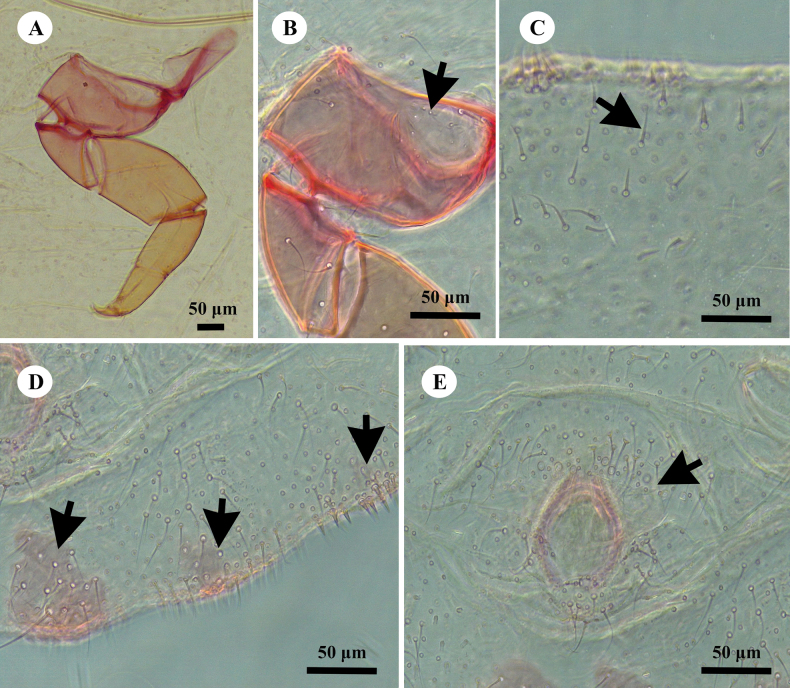
*Paraputoraufi* Zarkani & Kaydan, sp. nov., adult female holotype. A. Translucent pores present on hind coxa only; B. Setae flanking anal ring noticeably longer than other dorsal setae, and two pairs of long stout setae present just posterior to anal ring; C. Ventral sclerotized patches present on margins of abdominal segment VIII only, and cisanal setae slender; D. Vulva with a pair of noticeable sclerotized internal pockets.

***Slide-mounted adult female*** (values of holotype followed by range of 3 paratypes in parentheses) (Figs 5, 6). Body broadly oval, sometimes almost rotund, membranous, largest specimens 2.5 (2.5–2.9) mm long and 1.7 (1.7–2.2) mm wide. Anal lobes moderately developed, each ventral surface bearing a stout apical seta 84 (80–88) μm long arising from a large circular-to-oval sclerotized area, 76 (76–80) μm wide, on ventral margin of abdominal segment VIII, occupying much of lobe. Antennae each 390 (390–410) μm in total length, with 7 antennomeres; antennal setae mostly short. Individual antennal antennomere lengths (in μm) are as follows: antennomere I–VI, each 310 (310–320); and VII (apical antennomere), 90 (90–100) long and 30 (30–40) wide. Apical antennomere with 4 fleshy setae, each 50–55 μm long, and an apical seta 34 (34–38) μm long. Clypeolabral shield 345 (345–425) μm long and 388 (388–393) μm wide. Labium unusually long and pointed, longer than clypeolabral shield, 3 segmented, 210 (210–300) μm long, with basal segment 123 (123–128) μm wide. Anterior spiracles each 138 (138–143) μm long and 70 (70–75) μm wide across atrium; posterior spiracles each 138 (138–143) μm long and 70 (70–88) μm wide across atrium. Legs well developed; hind leg segments measuring (in μm): coxa, 307 (307–313); trochanter+femur, 378 (378–400); tibia+tarsus, 238 (238–253); and claw, 55 (55–70), without a denticle. Ratio of lengths of hind tibia+tarsus to trochanter+femur 0.63: 1; ratio of lengths of tibia to tarsus 1.4 (1.2–1.4): 1; and ratio of length of trochanter+femur to greatest width of femur 3.3 (3.3–3.8): 1. Hind leg with more than 300 translucent pores on coxa only (Fig. 6A). Tarsal digitules setose, each 53–60 μm long; claw digitules each minutely dilated distally, about 28–38 μm long. Anterior and posterior ostioles well developed, each containing a total of 62 (60–100) trilocular pores across both lips and 7 (5–9) setae. Circulus situated between abdominal segments III and IV, 155 (150–193) μm wide and divided by an intersegmental line. Anal ring about 100 (100–120) μm wide, situated on dorsum about 1.0–1.5 times its length from apex of abdomen; with 2 rows of cells and bearing 6 setae, each 75 (75–80) μm long. Cerarii numbering not distinct, with many intermediate conical setae, tending to merge. Anal lobe cerarii each normally containing 6 or 7 conical setae of different sizes, largest about 27.5 μm long and 6.3 μm wide at base, and a small compact group of trilocular pores. Anterior cerarii similar to anal lobe cerarii but with largest conical setae slenderer, and each cerarius often subdivided into 2 or 3 smaller cerarii: additional conical setae also present, so that cerarii appear to be continuous on some parts of margin.

***Dorsum*.** Slender, stiff setae present, mostly each about 12.5–20.0 μm long; long setae flanking anal ring fairly numerous, each 77.5–87.5 µm long, similar length to or shorter than an anal ring seta (each ring seta 75–100 µm long). Two pairs of long stout setae present just to posterior to anal ring, each about 105–113 µm long, longer and wider than an anal ring seta (Fig. 6B). Trilocular pores abundant. Discoidal pores fairly numerous, each slightly smaller than a trilocular pore.

***Venter*.** Normal flagellate setae present, mostly slender and each longer than a dorsal seta, 10–35 µm long. Ventral sclerotized patches present on anal lobes only. Cisanal and obanal setae slender, each 38–40 µm long, displaced to dorsal surface between anal ring and apex of abdomen. Multilocular disc pores each 10.0–12.5 μm in diameter, present on abdominal segments, distributed as follows (numbers): segment V: 3–5, VI: 31–38, VII: 30–32, and VIII: 8–10. Trilocular pores numerous but less abundant than on dorsum. Discoidal pores same size as on dorsum, scattered. Vulva becoming heavily sclerotized at maturity, 163–175 μm long and 88–100 μm wide; with a pair of noticeable sclerotized internal pockets, base of each 70–75 μm long and 30–38 μm wide (Fig. 6D). Oral collar tubular ducts of 3 sizes: (i) largest ducts each 10.0–12.5 μm long and 7.5 μm wide, distributed on abdominal segments (numbers): VII: 6–8, and VIII: 2–4; (ii) slightly smaller ducts, each 7.5–10.0 μm long and about 5 μm wide, varying in number, present posterior to vulva and across medial posterior edges of abdominal segments V–VIII; and (iii) minute ducts, each 7.5–8.0 μm long and about 2.5 μm wide, few, with 5 or 6 situated medially on abdominal segments V and VI.

#### Etymology.

The species is named after Prof. Dr. Aunu Rauf, an Indonesian entomologist who is interested in the conservation of native insects of Indonesia.

#### Host plants.

An unidentified forest tree (Meliaceae) (Fig. 4).

#### Distribution.

Indonesia (Kalimantan, East Kalimantan Province, Sepaku).

#### Comments.

*Paraputoraufi* is most similar to *P.spatholobi* Williams in possessing large oral collar tubular ducts present on margins of abdominal segment VI and ventral sclerotized patches absent from margins of abdominal segments VII and VI. However, *P.raufi* can be readily distinguished by having (character states for *P.spatholobi* given in parenthesis): (i) ventral sclerotized patches present on anal lobes (absent); (ii) multilocular disc pores present on abdominal segments V–VIII (present on abdominal segments VI–VIII); and (iii) vulva with a pair noticeable sclerotized internal pocket (without a pair of noticeable sclerotized internal pocket).

*Paraputoraufi* also resembles *P.latebrae* Williams in having: (i) large oral collar tubular ducts present on abdominal segment IV only, each duct over 1.5 times as wide as a trilocular pore, in distinct groups, even if only 1 or 2 present; (ii) circulus large, divided by an intersegmental line; and (iii) setae on each side of anal ring, of similar length to or a bit shorter than an anal ring seta, and ventral submarginal setae on abdominal segments V–VIII shorter than anal ring setae. However, *P.raufi* can be distinguished from *P.latebrae* in having (character states for *P.latebrae* given in parentheses): (i) setae flanking anal ring noticeably longer than other dorsal setae (setae flanking anal ring short, about same length as other dorsal setae); (ii) two pairs of long stout setae present just to posterior to anal ring (absent) (iii) ventral sclerotized patches present on margins of abdominal segment VIII only (ventral sclerotized patches present on margins of abdominal segments VII and VIII); (iv) cisanal setae slender, shorter than an anal ring seta (cisanal setae stout, similar in length to an anal ring seta); (v) translucent pores present on coxa only (translucent pores present on hind coxa and tibia, sometimes also on hind femur); and (vi) large oral collar tubular ducts absent from head (sometimes present on head).

It is also similar to *P.acehicus* Williams in having: (i) setae flanking the anal ring noticeably longer than other dorsal setae; (ii) two pairs of long stout setae present just to posterior to anal ring, all longer and wider than anal ring setae; (iii) cisanal setae slender, shorter than anal ring setae; (iv) translucent pores present on coxa only; and (v) large oral collar tubular ducts absent from head. However, *P.raufi* can be distinguished from *P.acehicus* in having (character states for *P.acehicus* given in parentheses): (i) large oral collar tubular ducts present (absent); (ii) ventral sclerotized patches present on margins of abdominal segment VIII (absent); and (iii) vulva with a pair of noticeable sclerotized internal pockets present (absent).

### ﻿Key to adult females of *Paraputo* found in Southeast and southern Asia (adapted from Williams (2004))

**Table:** 

1	At least 1 or 2 large oral collar tubular ducts present, each over 1.5 times as wide as a trilocular pore	**2**
–	Large oral collar tubular ducts (each over 1.5 times as wide as a trilocular pore) absent; either small ducts present, each as wide as a trilocular pore or smaller, or tubular ducts absent entirely	**20**
2	Large oral collar tubular ducts present on head, usually between antennal bases	**12**
–	Large oral collar tubular ducts absent from head, present on abdomen only	**3**
3	Large oral collar tubular ducts present between anal lobes	**4**
–	Large oral collar tubular ducts absent from between anal lobes	**5**
4	Large oral collar tubular duct groups on margins of abdomen present as far forward as abdominal segment II	***P.sinuosus* Williams**
–	Large oral collar tubular duct groups on margins of abdomen present on abdominal segment VII	***P.riedelae* Williams**
5	Circulus minute, about as large as a multilocular disc pore, not divided by intersegmental line. Large oral collar tubular ducts present on margins, occurring singly on abdominal segments VII, or VI and VII	***P.limitaneus* Williams**
–	Circulus larger and always divided by intersegmental line. Large oral collar tubular ducts on margins of abdomen present in distinct groups	**6**
6	Setae on each side of anal ring, longer than an anal ring seta. Ventral submarginal setae on abdominal segments VII–VIII mostly longer than an anal ring seta	***P.martini* Williams**
–	Setae on each side of anal ring, shorter than an anal ring seta. Ventral submarginal setae on abdominal segments V–VIII shorter than an anal ring seta	**7**
7	Large oral collar tubular ducts present on margins of abdominal segment VI	**8**
–	Large oral collar tubular ducts absent from margins of segment VI	***amydrus* Williams**
8	Ventral sclerotized patches absent from margins of abdominal segments VII and VI, cuticle in these positions membranous	**9**
–	Ventral sclerotized patches present on margins of abdominal segments VII and VI	**10**
9	Ventral sclerotized patches present on anal lobes. Multilocular disc pores present on abdominal segments V–VIII. Vulva with a pair of noticeable sclerotized internal pocket	***P.raufi* Zarkani & Kaydan, sp. nov.**
–	Ventral sclerotized patches absent from anal lobes. Multilocular disc pores present on abdominal segments VI–VIII. Vulva without a pair of noticeable sclerotized internal pocket	***P.spatholobi* Williams**
10	Ventral sclerotized patches present on margins of abdominal segments VI and VII	**11**
–	Ventral sclerotized patches present on margins of abdominal segment VII but absent from segment VI	***P.latebrae* Williams (in part)**
11	Dorsal setae each about 20–25 µm long. Legs stout, with translucent pores on hind coxa only. Oral collar tubular ducts present on abdominal segments VII and VIII. Multilocular disc pores present by posterior medial edge of abdominal segment VII	***P.martonoi* Zarkani & Kaydan, sp. nov.**
–	Dorsal setae each about 7.5 μm long. Legs slender, with translucent pores on hind coxa and tibia. Oral collar tubular duct situated on abdominal segments V–VIII. Multilocular disc pores present by posterior medial edges of abdominal segments VI–VIII	***P.carnosae* (Takahashi)**
12	Anal ring bearing more than 6 setae	**13**
–	Anal ring bearing 6 setae	**14**
13	Multilocular disc pores present on venter of abdomen	***P.mangiferae* (Betrem)**
–	Multilocular disc pores absent from venter of abdomen, except sometimes for a single pore	***P.corbetti* (Takahashi)**
14	Setae flanking anal ring short, about same length as other dorsal setae	**15**
–	Setae flanking anal ring noticeably longer than other dorsal setae	**16**
15	Abdominal segment VI with ventral margin sclerotized. Dorsal setae minute, mostly each 5 μm long, some scarcely longer than trilocular pores. Ventral oral collar tubular ducts present on abdominal segment IV	***P.capillulatus* Williams**
–	Abdominal segment VI with ventral margin membranous. Dorsal setae longer, mostly each 15–20 μm long. Ventral oral collar tubular ducts absent from abdominal segment IV	***P.latebrae* Williams**
16	Setae on each side of anal ring as long as anal ring setae or longer	***P. errabundus Williams***
–	Setae on each side of anal ring shorter than anal ring setae	**17**
17	Medial ventral abdominal oral collar tubular ducts each narrower than a trilocular pore	***P.sekayuensis* Williams**
–	Medial ventral abdominal oral collar tubular ducts noticeably wider than a trilocular pore, almost 1.5 times as wide	**18**
18	Ventral marginal groups of large-type oral collar tubular ducts present as far forward as abdominal segment II. Translucent pores present on coxae of middle legs in addition to those on hind coxae	***P.theaecola* (Green)**
–	Ventral marginal groups of large-type oral collar tubular ducts present only as far forward as abdominal segments V or VI. Translucent pores absent from coxae of middle legs, present on hind coxae only	**19**
19	Large-type oral collar tubular ducts present on margins of abdominal segments V–VIII. Medium-size ducts, each about 1.5 times as wide as a trilocular pore, present medially across abdominal segment IV	***P.malaccensis* (Takahashi)**
–	Large-type oral collar tubular ducts present on margins of abdominal segments VI and VII only. Medium-size ducts, each about 1.5 times as wide as a trilocular pore, absent from abdominal segment IV	***P.maschwitzi* Williams**
20	Oral collar tubular ducts present, each with deep collar occupying almost half length of duct. Anal ring always bearing more than 6 setae	**21**
–	Oral collar tubular ducts present or absent; when present, each with collar normal, narrow, around orifice only. Anal ring bearing 6 or more setae	**24**
21	Circulus absent. Multilocular disc pores on venter present as far forward as abdominal segment III	***P.malesicus* Willams**
–	Circulus present. Multilocular disc pores on venter present only as far forward as abdominal segments VI or VII, except for an occasional pore	**22**
22	Multilocular disc pores present only around vulva, absent from abdominal segment VI. Dorsal setae in medial areas of anterior abdominal segments about same length as surrounding setae, or shorter	***P.ranauensis* Williams**
–	Multilocular disc pores present around vulva and on segment VI. Some dorsal setae in medial areas of anterior abdominal segments noticeably longer than surrounding setae	**23**
23	Thoracic and abdominal segments I–IV with dorsal medial setae longer than surrounding dorsal setae	***P.cubicus* Williams**
–	Abdominal segments II and III only with dorsal medial setae longer than surrounding dorsal setae	***P.specicola* Williams**
24	Small oral collar tubular ducts entirely absent from venter	**25**
–	Small oral collar tubular ducts present on venter	**27**
25	Multilocular disc pores present on venter of abdomen only, around vulva. Long flagellate setae present on margins next to cerarii	***P.neonaucleae* Williams**
–	Multilocular disc pores numerous on venter of thorax as well as medial areas of abdomen. Long flagellate setae absent from margins next to cerarii	**26**
26	Circulus present. Discoidal pores each no larger than a trilocular pore	***P.areolatus* Williams**
–	Circulus absent. Discoidal pores each conspicuously larger than a trilocular pore	***P.drypetis* Williams**
27	Dorsal setae and trilocular pores present on medial areas of thorax and anterior abdominal segments. Ventral oral collar tubular ducts present on abdomen	**28**
–	Dorsal setae and trilocular pores absent from medial areas of thorax and anterior abdominal segments. Ventral oral collar tubular ducts absent from abdomen but present on medial areas of head and thorax	***P.lisponotus* Williams**
28	Medial oral collar tubular ducts absent from abdomen; only marginal ducts present	**29**
–	Medial oral collar tubular ducts present on abdomen	**30**
29	Ventral margins of abdomen membranous. Cerarii in 18 distinct groups, each with multiple conical setae. Marginal trilocular pores clustered within cerarii only. Oral collar tubular ducts present in marginal groups on abdominal segments VI and VII	***P.marlatti* Williams**
–	Ventral margins sclerotized, at least on abdominal segments VI–VIII. Cerarii, although sometimes distinct on abdomen, present in an almost continuous zone around head and thorax. Trilocular pores forming a noticeable compact zone around entire dorsal margin. Oral collar tubular ducts represented by 1 or 2 only on submargins of abdominal VII	***P.moogi Williams***
30	Oral collar tubular ducts present on head	**31**
–	Oral collar tubular ducts absent from head	**33**
31	Dorsal setae unusually long. Marginal oral collar tubular ducts present on head margin between antennal bases and on margins of thorax	***P.glycosmis* Williams**
–	Dorsal setae mostly short. Marginal oral collar tubular ducts absent from head margin between antennal bases and from margins of thorax	**32**
32	Venter of anal lobes membranous. Multilocular disc pores absent from abdominal segment V. Dorsal setae of 2 types; most dorsal setae anterior to abdominal segment VII short and clavate; marginal and submarginal setae mostly flagellate, longer than medial setae	***P.claviger* Williams**
–	Venter of anal lobes with quadrate sclerotized area. Multilocular disc pores on abdomen present on segment V and sometimes on segment IV. Dorsal setae all short and pointed, not differentiated in size	***P.banzigeri* Williams**
33	Ventral oral collar tubular ducts present in medial area posterior to vulva	**34**
–	Ventral oral collar tubular ducts absent from medial area posterior to vulva	**40**
34	Dorsal setae on head, thorax and anterior abdominal segments flagellate, stout, noticeably longer than conical cerarian setae. Clypeolabral shield with internal anterior extension	**35**
–	Dorsal setae on head, thorax and anterior abdominal segments either same length as conical cerarian setae or shorter. Clypeolabral shield without internal anterior extension	**36**
35	Dorsal setae on head and thorax noticeably shorter than setae on abdomen, each about 25–50 μm long	***P.sugonyaevi* Williams**
–	Many dorsal setae on head and thorax about same length as setae on abdomen, each about 100 μm long	***P.pandanicola* Williams**
36	Marginal groups of ventral oral collar tubular ducts present as far forward as abdominal segment IV. Translucent pores present on hind femora	***P.leveri* (Green)**
–	Marginal groups of ventral oral collar tubular ducts present only as far forward as abdominal segment VI. Translucent pores absent from hind femora	**37**
37	Sclerotized area on venter of each anal lobe large and quadrate, occupying most of lobe	***P.danzigae* Williams**
–	Sclerotized area on venter of each anal lobe smaller, elongate and slender	**38**
38	Ventral multilocular disc pores present as far forward as abdominal segment VI only. Dorsal setae on medial area of thorax and anterior abdominal segments noticeably stouter at base than setae around margins and posterior abdominal segments	***P.acehicus* Williams**
–	Vental multilocular disc pores present as far forward as abdominal segments IV or V. Most dorsal setae about same thickness at base	**39**
39	Cerarii distinct, with very few intermediate conical setae. Marginal trilocular pores, except those in cerarii, spaced similarly to others on dorsum. Ventral oral collar tubular ducts all about the same size. Most dorsal setae slender, each with width at base less than half as wide as a trilocular pore. Most ostiole setae each 15–20 μm long	***P.cribratus* Williams**
–	Cerarii less distinct, with many intermediate conical setae. Marginal trilocular pores present in a narrow band, more crowded than others on dorsum. Ventral oral collar tubular ducts of 2 sizes, with medial ducts smaller than marginal ducts. Most dorsal setal bases each at least half as wide as a trilocular pore. Most ostiole setae each about 40 μm long	***P.domatium* Williams**
40	Anal ring bearing more than 6 setae	**41**
–	Anal ring bearing 6 setae	**43**
41	All cerarii situated on ventral margins, even in teneral specimens. Anal lobe cerarii each situated on sclerotized area	***P.humboldtiae* Williams**
–	All cerarii situated on dorsal margins. Anal lobe cerarii each situated on membranous area	**42**
42	Anal lobes each with large ventral sclerotized area; other abdominal segments also with ventral sclerotized areas, sequentially smaller anteriorly. Cerarii compact, each normally containing 2 stout conical setae and slender auxiliary setae, except ocular cerarii (C), each sometimes with 3 conical setae. Circulus usually wider than long	***P.odontomachi* (Takahashi)**
–	Ventral margins of abdomen entirely membranous. Cerarii each containing multiple cerarian setae, occupying most of margin of each segment. Circulus usually longer than wide	***P.schellerichae* Williams**
43	Most dorsal setae on head, thorax and anterior abdominal segments long and flagellate, longer than cerarian setae	**44**
–	Most dorsal setae on head, thorax and anterior abdominal segments short and stiff, not noticeably longer than cerarian setae	**45**
44	Ventral oral collar tubular ducts present in marginal groups on abdominal segments VI and VII, present also in medial area of abdominal segment V only. Clypeolabral shield without inner anterior sclerotized extension	***P.riparius* Williams**
–	Ventral oral collar tubular ducts on abdominal margins represented by only 1 or 2 on segment VII, sometimes absent; present also in medial areas of abdominal segments V and VI. Clypeolabral shield with inner anterior sclerotized extension	***P.palmicola* Williams**
45	Marginal groups of ventral oral collar tubular ducts present on abdominal segments II–VII	***P.pahanensis* (Takahashi)**
–	Marginal groups of ventral oral collar tubular ducts present on margins of abdominal segments VI and VII only	**46**
46	Venter of each anal lobe with wide sclerotized area present. Setae beside anal ring noticeably longer than other dorsal setae. Clypeolabral shield without internal anterior extension	***P.ridleyi* (Takahashi)**
–	Venter of anal lobes membranous. Setae beside anal ring only slightly longer than other dorsal setae at most. Clypeolabral shield with internal anterior extension	***P.gigantochloae* Williams**

## Supplementary Material

XML Treatment for Paraputo

XML Treatment for Paraputo martonoi

XML Treatment for Paraputo raufi

## ﻿Additional information

### Use of AI

No use of AI was reported.

### Author ORCIDs

Agustin Zarkani https://orcid.org/0000-0001-9837-5019

Ariffatchur Fauzi https://orcid.org/0000-0001-9900-0614

Dwinardi Apriyanto https://orcid.org/0000-0002-5286-9010

Yahumri https://orcid.org/0000-0001-8509-1869

Rosfiansyah https://orcid.org/0000-0002-3868-6322

Mehmet Bora Kaydan https://orcid.org/0000-0002-0677-255X

### Data availability

All of the data that support the findings of this study are available in the main text.

## References

[B1] Ahmed AR Apori SO Karim AZ (2023) Mealybug vectors: A review of their transmission of plant viruses and their management strategies. AIMS Agriculture and Food 8(3): 736–761. 10.3934/agrfood.2023040

[B2] Ben-Dov Y (1994) A Systematic Catalogue of the Mealybugs of the World (Insecta: Homoptera: Coccoidea: Pseudococcidae and Putoidae) with Data on Geographical Distribution, Host Plants, Biology and Economic Importance. Intercept Limited, Andover, 686 pp.

[B3] Cox JM Pearce MJ (1983) Wax produced by dermal pores in three species of mealybug (Homoptera: Pseudococcidae). International Journal of Insect Morphology & Embryology 12(4): 235–248. 10.1016/0020-7322(83)90020-X

[B4] Daane KM Almeida RPP Bell VA Walker JTS Botton M Fallahzadeh M Mani M Miano JL Sforza R Walton VM Zaviezo T (2012) Biology and management of mealybugs in vineyards. In: Bostanian NJ Vincent C Isaacs R (Eds) Arthropod Management in Vineyards: Pests, Approaches, and Future Directions.Springer, Dordrecht, 271–307. 10.1007/978-94-007-4032-7_12

[B5] Danzig EM Gavrilov IA (2010) Mealybugs of the genera *Planococcus* Ferris and *Crisicoccus* Ferris (Sternorrhyncha: Pseudococcidae) of Russia and adjacent countries. Zoosystematica Rossica 19(1): 39–49. 10.31610/zsr/2010.19.1.39

[B6] Danzig EM Gavrilov-Zimin IA (2015) Palaearctic Mealybugs (Homoptera: Coccinea: Pseudo-coccidae). Part 2. Subfamily Pseudococcinae. Fauna of Russia and Neighbouring Countries, New series, No. 149: Insecta: Hemiptera: Arthroidignatha. Zoological Institute, Russian Academy of Sciences, St Petersburg, Russia, 619 pp.

[B7] Francardi V Covassi M (1992) Note bio-ecologishe sul *Planococcusnovae* (Nasonov) dannoso a *Juniperus* spp. in Toscana (Homoptera: Pseudococcidae). Redia 75: 1–20.

[B8] Franco JC Zada A Mendel Z (2009) Novel approaches for the management of mealybug pests. In: Ishaaya I Horowitz AR (Eds) Biorational Control of Arthropod Pests.Springer Science + Business Media, New York, 233–278. 10.1007/978-90-481-2316-2_10

[B9] García-Morales M Denno B Miller DR Miller GL Ben-Dov Y Hardy NB (2016) ScaleNet: a literature-based model of scale insect biology and systematics. http://scalenet.info [accessed 18 January 2023] 10.1093/database/bav118 PMC4747323 26861659

[B10] Joshi S Amarendra B Mendonce VP Sushil SN (2024) A new species of *Paraputo* Laing 1929 (Hemiptera: Coccomorpha: Pseudococcidae) from India. Zootaxa 5443(4): 567–579. 10.11646/zootaxa.5443.4.5 39645897

[B11] Kosztarab M Kozár F (1988) Scale Insects of Central Europe. Akadémiai Kiadó, Budapest, 456 pp. 10.1007/978-94-009-4045-1

[B12] Watson GW Muniappan R Shepard BM Sembel DT Xiong JJ Rauf A (2014) Sap-sucking insect records (Hemiptera: Sternorrhyncha and Thysanoptera: Thripidae) from Indonesia. The Florida Entomologist 97(4): 1594–1597. 10.1653/024.097.0432

[B13] Williams DJ (2004) Mealybugs of southern Asia. The Natural History Museum, London. Southdene SDN BHD, Kuala Lumpur, 896 pp.

[B14] Williams DJ Granara de Willink MC (1992) Mealybugs of Central and South America. CAB International, Wallingford, 635 pp.

[B15] Williams DJ Watson GW (1988) The Scale Insects of the Tropical South Pacific Region. Pt. 2. The Mealybugs (Pseudococcidae). CAB International, Wallingford, 260 pp.

[B16] Zarkani A Apriyanto D Turanli F Ercan C Kaydan MB (2021) A checklist of Indonesian scale insects (Hemiptera: Coccomorpha). Zootaxa 5016(2): 151–195. 10.11646/zootaxa.5016.2.1 34810456

[B17] Zarkani A Ercan C Apriyanto D Kaydan MB (2023a) Studies on mealybugs (Hemiptera: Coccomorpha) in Indonesia, with description of a new species and three new country records. Zootaxa 5228(2): 157–172. 10.11646/zootaxa.5228.2.4 37044657

[B18] Zarkani A Fauzi A Apriyanto D Kaydan MB (2023b) Mealybugs (Hemiptera, Coccomorpha, Pseudococcidae) on parasitic plants (Loranthaceae) in Indonesia with description of a new species and a new country record. ZooKeys 1167: 199–210. 10.3897/zookeys.1167.106012 37363737 PMC10288304

[B19] Zarkani A Watson GW Kaydan MB (2024a) A new species in the mealybug genus *Pseudococcus* Westwood (Hemiptera: Coccomorpha: Pseudococcidae) from Indonesia. Zootaxa 5555(4): 590–598. 10.11646/zootaxa.5555.4.6 40174034

[B20] Zarkani A Fauzi A Apriyanto D Kaydan MB (2024b) A new species of *Planococcus* Ferris (Hemiptera: Coccomorpha, Pseudococcidae) from Indonesia. Journal of Insect Biodiversity and Systematics 2(2): 231–242. 10.61186/jibs.10.2.231

